# chemtrain-deploy: A Parallel and Scalable Framework for Machine Learning Potentials in Million-Atom MD Simulations

**DOI:** 10.1021/acs.jctc.5c00996

**Published:** 2025-07-23

**Authors:** Paul Fuchs, Weilong Chen, Stephan Thaler, Julija Zavadlav

**Affiliations:** † Professorship of Multiscale Modeling of Fluid Materials, Department of Engineering Physics and Computation, TUM School of Engineering and Design, 9184 Technical University of Munich, 80333 Munich, Germany; ‡ Atomistic Modeling Center (AMC), Munich Data Science Institute (MDSI), 9184 Technical University of Munich, 85748 Garching, Germany; § 706370 Valence Labs, Montreal, QC H2S 3G6, Canada

## Abstract

Machine Learning Potentials (MLPs) have advanced rapidly and show great promise to transform Molecular Dynamics (MD) simulations. However, most existing software tools are tied to specific MLP architectures, lack integration with standard MD packages, or are not parallelizable across GPUs. To address these challenges, we present chemtrain-deploy, a framework that enables the model-agnostic deployment of MLPs in LAMMPS. chemtrain-deploy supports any JAX-defined semilocal potential, allowing users to exploit the functionality of LAMMPS and perform large-scale MLP-based MD simulations on multiple GPUs. It achieves state-of-the-art efficiency and scales to systems containing millions of atoms. We validate its performance and scalability using graph neural network architectures, including MACE, Allegro, and PaiNN, applied to a variety of systems such as liquid–vapor interfaces, crystalline materials, and solvated peptides. Our results highlight the practical utility of chemtrain-deploy for real-world, high-performance simulations and provide guidance for MLP architecture selection and future design.

## Graphical abstract

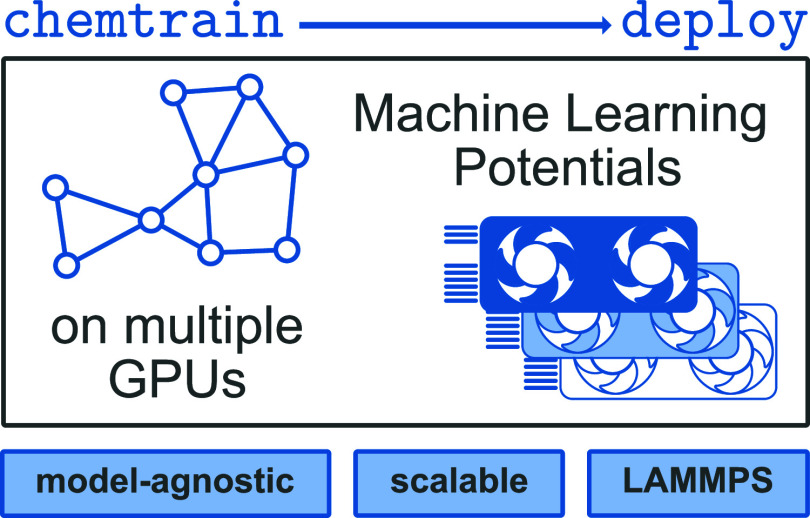

## 1 Introduction

In recent years, Machine Learning Potentials (MLPs) have advanced rapidly and found widespread applications in fields such as computational chemistry and materials science.^1–4^ By training the models on high-accuracy reference data sets, typically consisting of energies and forces, MLPs offer a promising compromise between accuracy and computational efficiency. They approach the accuracy of ab initio methods^5^ while maintaining computational speeds closer to classical force fields.^6–8^ This performance is achieved by capturing high-order, many-body interactions through either predefined^9,10^ or learned representations of local atomic environments,^11,12^ enabling near-linear scaling with system size^13^ and making them particularly attractive for large-scale simulations.

While there have been significant advancements,^14–16^ several challenges still hinder the widespread adoption of MLPs in real-world applications. From a model architecture perspective, Graph Neural Networks (GNNs) have emerged as a powerful approach due to their natural ability to encode atomic topologies and interactions.^15,17–22^ Numerous GNN architectures leveraging geometric priors have been proposed, with many accompanied by specialized software packages such as SchNetPack,^23^ TorchANI,^24^ MACE,^22^ TorchMD,^25^ and DistMLIP.^26^ However, these packages are often tightly coupled to their respective models and typically lack the modularity or plugin interfaces needed for seamless integration with widely used Molecular Dynamics (MD) software.^27–29^ This limits their extensibility and practical usability in domain-specific workflows.

Moreover, the rapid pace of innovation in other areas of the field presents additional challenges. New data curation strategies such as active learning,^30–32^ training methodologies,^33–35^ and schemes for incorporating long-range interactions^36–40^ are being developed continuously. Integrating these advancements into existing software frameworks often demands additional engineering effort, which can lead to a more diverse and specialized ecosystem. This fragmentation makes it harder for practitioners to adopt state-of-the-art methods and for developers to maintain robust, general-purpose tools.

Another emerging concern is how MLP performance is evaluated. While most efforts have focused on minimizing force and energy prediction errors, recent studies argue that simulation stability and the accuracy of observable quantities are more critical for practical applications.^41–45^ Benchmarking different architectures against specific simulation tasks is thus gaining importance, as it provides insights that are more relevant for real-world use and future model development.

In response to these challenges, several projects have attempted to provide easy-to-use interfaces between the ML core and different types of traditional modeling software. Plugins have been developed, such as Allegro-LAMMPS,^18^ SevenNet,^46^ GROMACS-NNpot,^28^ FitSNAP,^47^ OpenMM-Torch,^48^ and DeePMD-kit^49^ to enable simulations with MD software such as LAMMPS,^29^ GROMACS,^28^ and OpenMM.^48^ DeePMD-kit has previously demonstrated strong scalability for specific non-GNN architectures,^50^ but its recent extensions by a multibackend framework^51^ and added support for external models^52^ have not been demonstrated to scale to multi-GPU systems. Thus, existing solutions are constrained by either architecture-specific designs or limited parallelizability.

In this work, we present chemtrain-deploy, a model-agnostic deployment framework that extends our existing JAX-based training platform, chemtrain.^53^ chemtrain was originally designed to support customizable training of neural network potentials with different training strategies, which integrates with JAX, M.D^54^ to offer a unified training and simulation environment. However, current JAX, M.D. lacks the robustness, interoperability, and scalability of established packages such as LAMMPS. chemtrain-deploy bridges this gap by enabling seamless deployment of pretrained semilocal MLPs into LAMMPS, allowing efficient large-scale simulations with systems containing millions of atoms across multiple GPUs. To demonstrate flexibility and scalability, we benchmark chemtrain-deploy on three widely used GNN models: MACE, Allegro, and PaiNN, all trained to comparable accuracy. We test them on diverse systems, including water–vapor coexistence, solid state (fcc) aluminum, and solvated Chignolin, evaluating strong and weak scaling, parallel efficiency, and performance relative to other frameworks such as JAX, M.D. Our results demonstrate the practicality of chemtrain-deploy for real-world, high-performance molecular simulation and provide guidance on GNN selection and future development.

## 2 (Semi-)Local Potential Models

MD simulations can describe the behavior of atomistic systems through forces ***f*** that are the negative gradients of a potential energy function $f_{i}=-\frac{\partial U\left(r\right)}{\partial r_{i}}$ . In many systems, atoms interact predominantly with other atoms in their local environment. Therefore, many classical and modern models approximate the total energy of a system through a sum of local atomic energy contributions

$$U\left(r\right)=\overset{N}{\underset{i=1}{\sum }}U_{i}\left(r_{i},A_{i}\right)$$ 1

where $A=\left\{\left(r_{j},Z_{j}\right)|j\in N_{=1}\left(i\right)\right\}$ is the set of atom positions and species *Z*_*i*_ of the direct neighbors $N_{=1}\left(i\right)$ to particle *i* with a distance ∥ ***r***_*ij*_∥ less than a cutoff.^55,56^

### 2.1 Descriptors

The predicted potential energy should be invariant to permutations of atoms of the same species and translation, rotation, and reflections of the reference frame.^55,56^ However, applying general regression models such as Neural Networks or Kernel Models directly to the atomic positions does not necessarily result in a model that respects these invariances.^55^ Therefore, many potential models first encode the atomic environments into an invariant vector of local descriptors $\xi =\left\{\xi^{\alpha }\left(r_{i},A_{i}\right)\right\}$ that serve as input to an learnable atomic energy function $U_{i}\left(r,A_{i}\right)={Ũ}_{i}\left(\xi_{i}\left(r_{i},A_{i}\right)\right)$ ^56^ such as a Neural Network.^55^ Using the same model and descriptors for all particles of the same species ensures geometric and permutation invariant potential predictions.^55^

Local descriptors typically achieve translational invariance by acting on the atom displacements ***r***_*ij*_ rather than the absolute positions.^56^ Moreover, descriptors such as Atom-Centered Symmetry Functions (ACSF) achieve rotation and reflection invariance by encoding distances and directions between pairs and triplets of neighbors through invariant distances and angles. However, this encoding scales unfavorably with the number of neighbors for more than two-body correlations. Therefore, equivariant descriptors such as the Smooth Overlap of Atomic Positions (SOAP)^57^ represent displacements in a suitable basis and perform equivariant operations to encode correlations between multiple neighbors while scaling linearly with the number of neighbors. More generally, the Atomic Cluster Expansion (ACE) provides a systematic approach to constructing a complete basis for the local environment through hierarchical expansion. Thus, the ACE descriptor can represent many previously proposed local descriptors while scaling linearly with the number of neighbors.^12^

### 2.2 GNNs

Devising accurate and efficient descriptors by hand for chemically diverse data sets is difficult. Approaches such as ACE describe how to systematically build a complete basis for the local environment.^56^ However, the resulting descriptors scale unfavorably with the number of atom species due to the number of basis functions.^18^ Thus, learning efficient environment descriptors from data through GNNs has gained wide attention.

GNNs encode locality by representing the system as a graph, with nodes representing atoms and edges connecting all neighbors $N_{=1}$ . The graph is commonly embedded by assigning node features *h*_*i*_^(0)^ = *f*_h_(*Z*_*i*_) based only on the particle species to ensure permutation invariance and edges features *e*_*ij*_^(0)^ = *f*_e_(***r***_*ij*_) based on edge displacements to ensure translational invariance. Many proposed models, such as SchNet^20^ or DimeNet,^58^ then extract environment descriptors through the Message-Passing (MP) framework^11^ by propagating information along the graph through messages

$$m_{i}^{t+1}=\underset{j\in N\left(i\right)}{\sum }M^{t}\left(h_{i}^{t},h_{j}^{t},e_{ij}\right)$$ 2

which aggregate information from neighboring atoms by a learnable message function $M^{t}$ . Using the aggregated messages, the MP-GNNs then update the node features

$$h_{i}^{t+1}=U^{t}\left(m_{i}^{t+1},h_{i}^{t}\right)$$ 3

through an update function $U^{t}$ . The final node features ***h***_*i*_^*T*^ after *T* message passing steps can act as input to a regression model such as a Neural Network^20^ or a Gaussian Process.^59^

Similar to classical descriptors, the GNN predictions must be invariant with translations, rotations, and reflections. Early GNNs achieved this invariance through an invariant graph embedding using distances^20^ and angles.^58^ However, higher-order embeddings can improve the GNN expressiveness but scale unfavorably with the number of neighbors,^18^ similar to ACSFs. Moreover, propagated messages contain only invariant information about the particle’s local environment, prohibiting leveraging information about their relative orientation.^21^ Therefore, equivariant MP-GNNs generalize message-passing to tensorial features, such as displacements between atoms, to efficiently propagate directional information about atomic environments. Thereby, equivariant GNNs employ operations that ensure tensorial features are equivariant, i.e., transform similarly to the input for a group of transformations, to ensure invariance of the final scalar node features.^17,19,21^

Unlike the previously described strictly local descriptors, messages passing GNNs can propagate information between atoms that are not direct neighbors. Instead, GNNs propagate information from an atom *i* to higher-order neighbor atoms $N_{\leq T}$ connected by a path of length less or equal than *T*. Thus, MP-GNNs with many message-passing layers have a large receptive field radius *TR*, potentially impairing their scalability. To ensure high scalability, multiple approaches aim at constructing descriptive GNNs with small receptive fields. The Multi-ACE framework^17^ reformulates message construction in the ACE formalism, generalizing previous invariant and equivariant MP-GNNs, such as SchNet,^20^ DimeNet,^58^ NequIP,^19^ and PaiNN,^21^ that correlate only information from a limited number of neighbors for each message. Through the ACE formalism, this framework enables models such as MACE^22^ to construct messages that correlate information from an arbitrary number of neighbors to exploit high-order many-body correlations independently of the number of message-passing steps while scaling linearly with the number of neighbors. The Allegro model^18^ reformulates message-passing in an edge-centric formalism. Therefore, Allegro passes messages only between directed edges originating from the same node. Consequently, no information is propagated to particles outside the cutoff shell. Consequently, the Allegro model learns strictly local environment descriptions.

### 2.3 Chosen Architectures

In this work, we chose the GNN models PaiNN, MACE, and Allegro as examples of different design choices of models in terms of receptive fields and fidelity of semilocal descriptions. On the one hand, the PaiNN model correlates pairs of neighbor features in only each message-passing layer, such that the number of message-passing layers determines the receptive field and the body order of the final descriptor. On the other hand, the Allegro model employs equivariant edge-based message passing to ensure strict locality, such that the number of message-passing layers only affects the body order but not the receptive field of the model. The MACE model employs the ACE formalism to correlate information from a variable number of neighbors. Therefore, the final body order can be enlarged without increasing the receptive field.

### 2.4 Model Training

We trained all models using a force-matching approach.^53,60^ Given a data set of atomic configurations with reference energies *U* ^ref^ and reference forces ***f*** ^ref^, we optimize the neural network parameters θ to minimize differences between predicted and reference values. Thus, the training loss for *S* number of samples is

$$L(\theta )=\frac{\lambda_{E}}{S}\overset{S}{\underset{\alpha =1}{\sum }}|U^{\theta }(r_{\alpha },Z_{\alpha })-U_{\alpha }^{\mathrm{ref}}{|}^{2}+\frac{\lambda_{F}}{S}\overset{S}{\underset{\alpha =1}{\sum }}\left[\frac{1}{3N_{\alpha }}\overset{N}{\underset{i=1}{\sum }}∥f_{i}^{\theta }(r_{\alpha },Z_{\alpha })-f_{\alpha ,i}^{\mathrm{ref}}{∥}^{2}\right]$$ 4

where λ_E_ and λ_F_ control the relative weight of the energy and force terms.

## 3 Software Description

### 3.1 Structure of chemtrain-deploy

chemtrain-deploy complements the chemtrain^53^ framework to apply trained models in large-scale MD simulations using established MD software. Therefore, chemtrain-deploy comprises three parts: exporting a trained model, importing it into established MD software through a plugin or modification, and efficiently evaluating the model on high-performance hardware. The parts and workflow of chemtrain-deploy are depicted in Figure 1.

**Figure 1:**
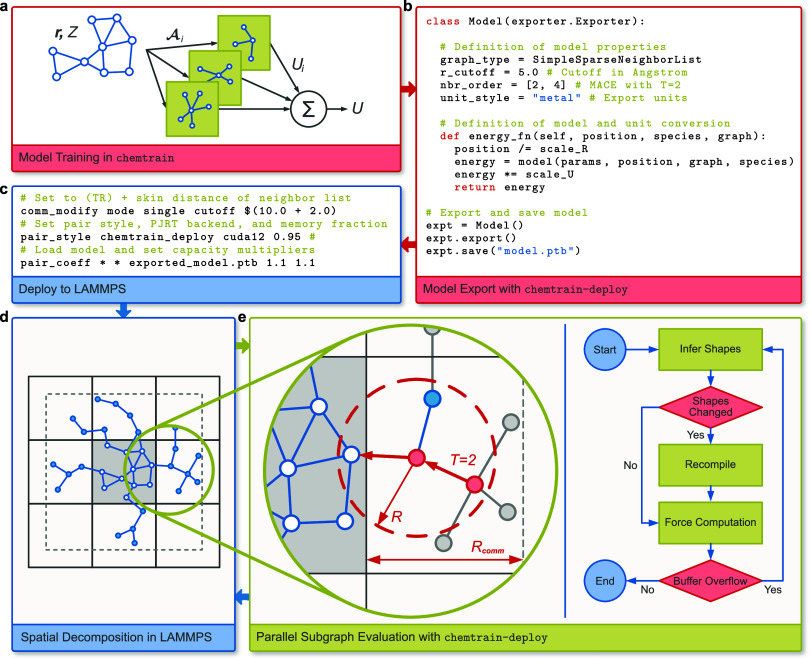
Overview of chemtrain-deploy **.** Model trained in chemtrain (a) is exported (b) and loaded into LAMMPS (c). LAMMPS distributes the workload onto multiple processors by decomposing the system into domains (solid lines) and computes domain neighbor lists including atoms in the domain (blue empty) and atoms from other domains (blue filled) within *R*_comm_ ≥ *TR* (dashed gray lines) (d). chemtrain-deploy computes the potential and forces independently for each domain, pruning atoms (gray) from the neighbor list graph generated by LAMMPS and buffering atom and graph data to fixed shapes required by XLA (e).

First, chemtrain-deploy extends the framework chemtrain (Figure 1a) to export trained models to a self-contained format (Figure 1b). The export saves the model architecture and parameters through the MLIR framework^61^ and properties that define the input and output of the model, such as length and energy units, the maximum length of graph edges, and the format of the input graph. Therefore, the exported model file contains all of the information needed to apply the model and is thus simple to share or archive within the JAX compatibility guarantees.

Second, chemtrain-deploy consists of a plugin to load and use the exported model for large-scale MD simulations in established MD software (Figure 1c). The MD software provides basic and advanced algorithms to perform MD simulations. Moreover, MD software provides algorithms to decompose the system into multiple domains for parallelization and create a neighbor graph representation of the system (Figure 1d). Therefore, the plugin interfaces chemtrain-deploy with framework-specific algorithms for parallelization and to run advanced MD simulations with the exported model. In the current version, chemtrain-deploy provides a plugin to the MD software LAMMPS.^29^

Finally, chemtrain-deploy provides a library for the plugin to evaluate the exported model (Figure 1e). This library uses XLA^62^ and PJRT^63^ to translate the model into an efficient backend-specific computation at runtime. Therefore, the library transforms and buffers the MD software’s atom and neighbor data for compilation with XLA. Following, the XLA compiler performs hardware-independent optimizations such as common subexpression elimination and operation fusion to reduce computational cost and memory requirements. Finally, the pluggable PJRT runtimes further optimize the code for specific backends, such as GPUs and CPUs, considering the backend’s architecture. Thus, the library extends LAMMPS’ capabilities to run efficiently on specific hardware and evaluate new force-field architectures without rewriting or recompiling LAMMPS. Moreover, the shared library promotes future extension of chemtrain-deploy by reusing provided functionality in new plugins, respectively extensions, to other MD software.

### 3.2 Distributed Potential Computation

In the following, we describe in more detail how chemtrain-deploy approaches distributed potential and force computation for semilocal potentials defined in eq 1 (see Section 2). Given a graph of the system, which represents atoms by nodes that share edges if closer to each other than a cutoff distance *R*, the local environment $A_{i}=\left\{\left(r_{j},Z_{j}\right)|j\in N_{\leq T}\left(i\right)\right\}$ contains positions ***r***_*j*_ and species *Z*_*j*_ of all atoms that are direct neighbors $N_{=1}\left(i\right)$ of node *i*. For semilocal potential models such as message-passing GNNs with *T* message-passing steps, the local environment additionally contains atoms *i*, referred to as *T*-th order neighbors $N_{\leq T}\left(i\right)$ , to which a path of at most length *T* exists (see Figure 1e).

chemtrain-deploy computes the potential energies in parallel on multiple independent processors (GPUs/CPUs) by partitioning the full graph into one subgraph per processor using spatial system decomposition and neighbor list generations provided in MD software, e.g., LAMMPS.^29^ As outlined in Figure 1d, LAMMPS distributes the workload by dividing the system into nonoverlapping domains, such that each atom is local to exactly one domain. LAMMPS then assigns the domains and the contained local atoms to the available processors. For each processor, LAMMPS additionally copies all atoms from other domains within a distance of *R*_comm_ of the processor’s domain boundary and constructs a neighbor list graph. Since the maximum distance between an atom *i* and any of its *T*-th neighbors can be *TR*, choosing *R*_comm_ ≥ *TR* ensures that the neighbor graph of each domain contains all *T*-th neighbor atoms of all local atoms. Thus, summing up the predicted energies of local atoms results in the total potential energy of the system, as each atom energy *U*_*i*_ is computed exactly once on a subgraph with a complete environment of *i*.

Running MD simulations requires chemtrain-deploy to compute the forces acting on the atoms. From the sum rule, the total force on an atom

$$f_{i}=-\frac{\partial U_{i}\left(r_{i},R_{i}\right)}{\partial r_{i}}-\underset{j\in N_{\leq T}^{R}}{\sum }\frac{\partial U_{j}\left(r_{j},R_{j}\right)}{\partial r_{i}}$$ 5

decomposes into a sum of partial forces $f_{ij}=-\frac{\partial U_{j}\left(r_{j},R_{j}\right)}{\partial r_{i}}$ . The total forces of all local atoms can be computed directly on each processor by computing all nonzero partial forces. However, this approach generally requires extending the domain subgraph to include the 2*T*-th order neighbors, which are necessary to correctly compute the potential energy of all *T*-th order neighbors of the local atoms. Alternatively, chemtrain-deploy computes the partial forces of all local atoms with respect to all atoms of the domain subgraph. Then, the total forces on each local atom can be obtained by summing all partial forces from the corresponding copies on other processors. Thus, by constructing graphs containing all *T*-th order neighbors of local atoms, all particles’ forces and potential energies can be computed with initial and final but without intermediate communication operations.

The graph obtained from LAMMPS might not be minimal and may contain copied atoms that are not within the semilocal environment of any local atom (gray nodes and edges in Figure 1e). Moreover, neighbor lists are typically constructed with edges longer than the model cutoff to prevent a costly neighbor list recomputation at every time step. Therefore, chemtrain-deploy prunes the neighbor list graph at every time step to improve the costly model evaluation. First, chemtrain-deploy removes all edges from the graph that are longer than the specified cutoff. Following chemtrain-deploy identifies all *T*-th neighbors of the local atoms by sending out pseudomessages from the local atoms. Each atom that received messages in the previous steps sends a message in the next step. Finally, after the *T* message passing steps, all *T*-th neighbors of local atoms received a message. chemtrain-deploy automatically adds the pruning computation to the model during export (Figure 1b). Therefore, pruning operations are parallelized and optimized through XLA.

### 3.3 Parallelization Cost

We estimate the cost of parallelizing semilocal potential models in homogeneous systems. In homogeneous systems, the cost of a semilocal potential model scales linearly with the number of atoms in the domain.^18^ We assume that the system domains are rectangular boxes with side lengths *L*_*x*_ = *L*_*y*_ = *L*_*z*_ = *L* for bulk systems periodic in all dimensions and *L*_*x*_ = *L*_*y*_ = *L* ≫ *L*_*z*_ for surface systems periodic in the *x* and *y* dimension, the total number of atoms, *N*, is proportional to *L* ^*d*^, where *d* is the number of periodic dimensions. However, due to copied particles within a distance of *TR* to the domain boundary, the cost of computing energies and forces is proportional to (*L* + 2*TR*)^*d*^.

The workload can be divided among *P* processors by using the domain decomposition described above to accelerate the computation. Therefore, each processor computes forces and energies for a domain of approximately length *P* ^–1/*d*^ *L* in the periodic dimension, still requiring copies of atoms within *TR* distance to the domain boundary. Under the assumption that the runtime is proportional to the cost, the parallelization speeds up the computation by a factor

$$S={\left(\frac{L+2TR}{P^{-1/d}L+2TR}\right)}^{d}$$ 6

Since *P* processors have to spend a relatively higher amount of work on computing interactions between copied atoms than between local atoms, the total work increases, causing a decrease in the parallel efficiency

$$\varepsilon =\frac{S}{P}$$ 7

### 3.4 Runtime Optimizations and Buffering

chemtrain-deploy optimizes and evaluates the model through XLA. However, XLA reoptimizes the program every time the shape of an input changes, which typically requires more time than the actual computation. Thus, chemtrain-deploy buffers all dynamically shaped inputs to a fixed shape and evaluates the model as outlined in Figure 1. First, chemtrain-deploy computes the required buffer shapes. These shapes can vary, for example, if the number of atoms and neighbors in the domain changes. If the buffer capacities are exceeded, then chemtrain-deploy enlarges the buffers and recompiles the model. Therefore, chemtrain-deploy leverages the call module loader from the TensorFlow project,^64^ based on XLA, to transform the exported model into XLA operations, e.g., by concretizing buffer shapes. If no buffer overflowed, chemtrain-deploy transforms and copies data to the device and performs the computation. The models use internal buffers to enable optimizations, such as graph pruning, in the computation. Thus, after the computation, chemtrain-deploy checks whether internal model buffers overflowed. If internal model buffers overflow, chemtrain-deploy repeats the computation with resized model buffers. If no buffer overflowed, chemtrain-deploy copies back the computed forces and returns statistics of the computation.

Since chemtrain-deploy only enlarges buffers, the frequency of recompilations decreases for systems at equilibrium. However, recompilations can happen frequently in the initial stages of computations on multiple devices if each device recompiles independently. Thus, chemtrain-deploy enforces the collective recompilations of multiple devices per time step by explicitly controlling recompilations. Therefore, the chemtrain-deploy plugin first attempts to evaluate the model with recompilation disabled. If a recompilation is necessary on one device, the device raises an exception that will be caught by the plugin. The plugin then synchronizes the error to all devices, which will enlarge overflown and nearly filled buffers and recompile the program. Thereby, chemtrain-deploy boosts simultaneous recompilations on multiple devices to shorten warm-up periods in large-scale parallel applications.

## 4 Results

### 4.1 Benchmark Systems and Models

We benchmarked our framework on three chemically and structurally diverse systems commonly studied in biophysics and materials science: a liquid–vapor water system, a crystalline aluminum solid, and the miniprotein Chignolin solvated in water. These systems span homogeneous and heterogeneous environments and include different phase states, such as liquid, solid, and interfacial configurations, capturing a broad range of chemical and structural complexity. For the aluminum case, a face-centered cubic lattice (lattice constant 4.065 Å) was constructed and replicated equally in all three dimensions; simulations were conducted at 1000 K with a 3 fs time step and a 2.0 Å neighbor skin. For the Chignolin case, the system was read from a preconfigured structure created by GROMACS, consisting of a Chignolin molecule solvated in a 3.3 nm cubic TIP3P water box. The system was then replicated equally in all three spatial dimensions, according to the scaling index. Simulations were performed at 293.15 K by using a 0.5 fs time step and a 2.5 Å neighbor skin distance. For the water–vapor interface case, the system was initialized from a pre-equilibrated 2 × 2 × 5 nm^3^ TIP3P water box and replicated equally in the *x* and *y* directions according to a scaling index; the *z*-direction was extended to create vacuum regions for a water–vacuum interface, similar to the prior setup.^65^ This simulation was run at 293.15 K using a 1 fs time step and a 2.5 Å neighbor skin distance.

For each system, we chose a corresponding training data set: H2O-PBE0TS,^13^ ANI-AL,^31^ and SPICE,^66^ respectively. We used three different GNN architectures that reflect the methodological diversity of modern approaches: Allegro,^18^ MACE,^22^ and PaiNN,^21^ on the same data set for each system. All models were trained using single-precision (FP32) arithmetic and the Adam optimizer with the following default parameters: β_1_ = 0.9, β_2_ = 0.999, and ϵ = 10^–8^. For each data set, a consistent train–test–validation split ratio of 7:2:1 was used, where we used the validation split to select the best performing parameters. All models use a graph cutoff distance of 5 Å. For fair comparison, we carefully selected hyperparameters to achieve similar energy and force accuracy across architectures. In all cases, the models reached comparable MAE or RMSE values, remained within chemical accuracy, and matched reported literature benchmarks (Table 1). Detailed architecture-specific hyperparameters are provided in the Supporting Information.

**Table 1:** Root Mean Square (RMSE) and Mean Absolute Errors (MAE) for Energies (meV/atom) and Forces (meV/Å) across Allegro, MACE, and PaiNN Models on the ANI-AL, SPICE, and H_2_O-PBE0TS Datasets, with Reference Values from the Literature, Including Classical MEAM and Other MLPs

	Allegro	MACE	PaiNN	reference
**ANI-AL**				ANI-AL,^31^ MEAM^67^
energy (RMSE)	12.9	9.9	7.2	1.9, 60.6
force (RMSE)	109.4	71.8	62.7	60.0, 244.8
**SPICE**				TorchMD-NET^66^
energy (MAE)	12.4	46.1	27.4	48.3
forces (MAE)	73.5	47.6	48.4	
**H**_2_ **O-PBE0TS**				NequIP,^19^ DeepMD^13^
energy (RMSE)	0.7	0.5	0.7	0.6, 0.3
force (RMSE)	36.2	13.3	17.2	11.6, 40.4

### 4.2 Memory Requirements

GNN-based MLPs can require significant memory to store high-dimensional node features and messages. However, model-agnostic software such as JAX, M.D., GROMACS-NNPot, or OpenMM-Torch does not support multi-GPU simulations through domain decomposition. Therefore, we determined the maximally supported system sizes and runtimes for MD simulations using GNN potentials that can be run on a single GPU with JAX, M.D. Additionally, we report reference measurements for chemtrain-deploy, which, unlike JAX, M.D., is not limited to only one GPU.

We tested all combinations of models and systems for which we report accuracies in the previous section. To increase the system sizes, we replicated all systems equally in all periodic dimensions, as described above. We performed MD simulations in the NVT ensemble using a Nosé–Hoover thermostat in LAMMPS with the chemtrain-deploy pair style implemented in our custom chemtrain-deploy interface and in JAX, M.D. Each simulation was run for 100 equilibration steps followed by 250 production steps on a single A100 GPU with 80GB of memory.

For JAX, M.D., the maximum system sizes were lower than those for chemtrain-deploy (Table 2), limited to less than half a million atoms. In comparison, chemtrain-deploy could simulate more or a similar number of atoms than JAX, M.D. with the strictly local Allegro model on one GPU. Differently, for the message-passing models, the maximum system sizes that could be simulated with chemtrain-deploy were lower than those for JAX, M.D. In all cases, the runtime chemtrain-deploy was similar or slower than that for JAX, M.D. (Supporting Information, Table S1).

**Table 2:** Maximum System Sizes in Number of Atoms for JAX, M.D. vs chemtrain-deploy on a Single GPU (A100, 80GB) for Allegro, MACE, and PaiNN Applied to Solid State Aluminum (fcc) at 1000 K, Replicated Box of Solvated Chignolin at Ambient Conditions, and Water Slab at Ambient Conditions

	system	JAX, M.D.	chemtrain-deploy
Allegro	aluminum	296,352	470,596
	Chignolin	27,936	27,936
	water	253,125	496,125
MACE	aluminum	202,612	108,000
	Chignolin	94,284	94,284
	water	162,000	112,500
PaiNN	aluminum	340,736	171,500
	Chignolin	27,936	3,492
	water	72,000	40,500

The difference in memory consumption and computational efficiency is likely due to JAX, M.D. updating the neighbor list entirely on the GPU and applying periodic boundary conditions without copied atoms. For large systems and short-ranged models, the neighbor list generation can require more memory in JAX, M.D. than computing interactions for copied atoms in chemtrain-deploy. For smaller systems and models with larger effective cutoffs, memory and compute requirements for copied atoms are higher than for local atoms, affecting the computational efficiency (see Supporting Information, Figure S1). However, due to the copied atoms, chemtrain-deploy can parallelize the simulation on multiple GPUs. Therefore, using additional GPUs could compensate for the higher memory requirements. In contrast, JAX, M.D does not support parallelization, such that the memory requirements limit the maximum system sizes below the order of a million atoms. However, applications such as the investigation of solidification can still exhibit finite-size effects up to two million atoms.^68^ Thus, the single-GPU support prevents software such as JAX and M.D. from deploying GNN potentials to applications requiring large-scale simulations.

### 4.3 Scaling to Million-Atom Systems

To estimate the performance of chemtrain-deploy for simulating large systems on multiple GPUs, we evaluated strong and weak scaling for all combinations of systems and models. These scaling simulations were conducted on the JEDI test system using up to 16 nodes interconnected via an InfiniBand NDR200. Each node consists of 4 NVIDIA GH200 Superchips, with each Superchip pairing 72 CPU cores and an H100 GPU with 96GB of memory. For each combination, we selected a different system size to respect the memory requirements of the models and ran MD simulations, as described for the previous experiment.

For the strictly local Allegro model, we observed close-to-ideal strong scaling (Figure 2), slightly outperforming the anticipated strong scaling in eq 6 and often exceeding the anticipated ideal parallel efficiency (Supporting Information, Figure S2). This improvement might be due to XLA optimizations leveraging additional memory and compute resources, which is not accounted for by the linear relation between the runtime and number of atoms anticipated in eq 6. For the message-passing GNNs, we obtained good strong scaling, except for Chignolin simulated with the PaiNN model. In all cases, the measured strong scaling is consistent with our approximation given in eq 6 for all systems.

**Figure 2:**
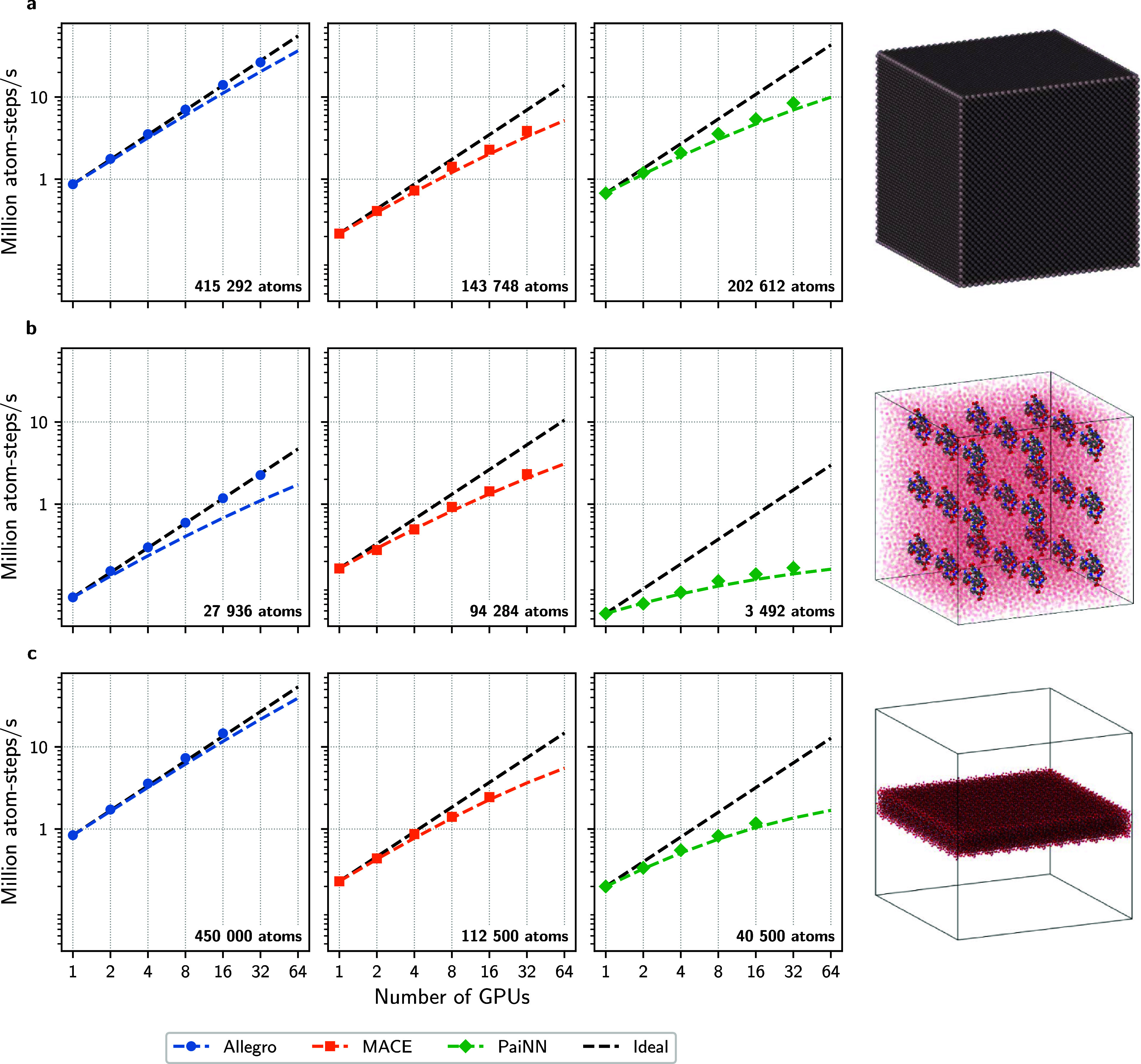
Strong scaling on JEDI for Allegro (blue), MACE (orange), and PaiNN (green) applied to (a) solid state aluminum (fcc) at 1000 K, (b) replicated box of solvated Chignolin at ambient conditions, and (c) water slab at ambient conditions next to visualizations of the systems. The sizes of each system are given in numbers of atoms in the bottom right corner. Visualizations were created with OVITO^69^ for the systems corresponding to the MACE model. Ideal strong scaling, based on a linear relation between runtime and number of processors, and approximate (eq 6) strong scaling, based on a linear relation between runtime and total number of atoms per processor, are shown as dashed lines in black and the model colors, respectively.

As shown in Figure 3, all models exhibited a close-to-ideal weak scaling. This result indicates that interdevice and internode communications do not crucially affect efficiency for multi-GPU computations. Therefore, scaling in chemtrain-deploy is mostly determined by the effort spent on copied atoms compared to local atoms (Supporting Information, Figure S1). Thus, eq 6 provides a good reference to estimate the required cost and resources of scaling message-passing GNNs to large systems.

**Figure 3:**
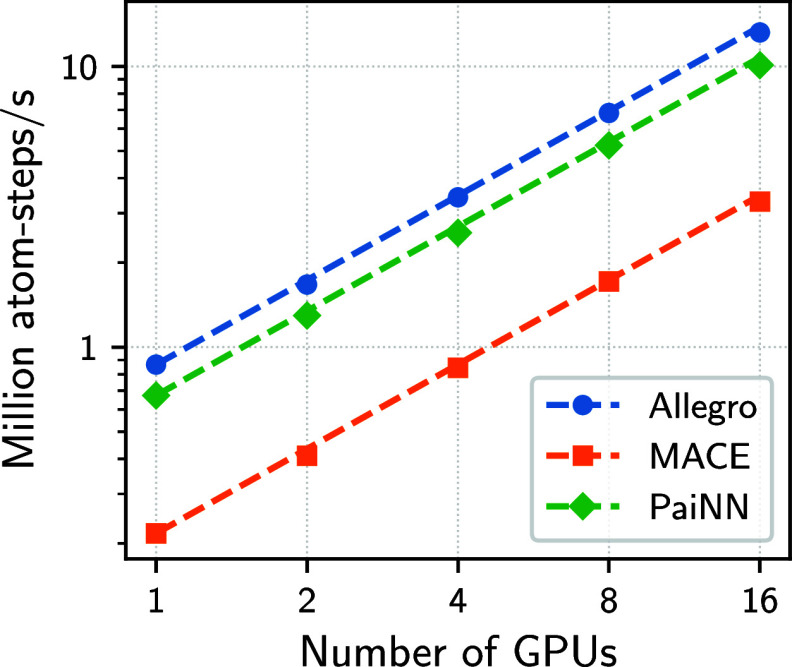
Weak scaling on JEDI for Allegro (blue), MACE (orange), and PaiNN (green) applied to solid state aluminum (fcc) systems at 1000 K with 415, 292, 143, 748, and 202, 612 atoms per GPU. Ideal weak scaling is displayed as dashed lines for each model in the respective color.

To directly compare the models, we also evaluated the scaling on million-atom systems, visualized in Figure 4 (simulation speeds reported in Supporting Information, Table S2). The Allegro and MACE models showed good strong scaling for all systems. The PaiNN model scaled slightly worse than the other models in the aluminum system and failed for the Chignolin and water systems due to insufficient memory. Comparing the simulation speed of all models, the Allegro model performed best for aluminum and water. The MACE models achieved a similar throughput for all systems, outperforming the speed of Allegro for the Chignolin system. The PaiNN model performed similarly to the Allegro model in the aluminum system. Based on these scaling results, we conclude that the Allegro model is highly efficient for systems with a few atom types, such as water and aluminum. However, for chemically diverse systems, MACE provides a better trade-off between accuracy, scalability, and robustness (Supporting Information, Section 2.1). Using PaiNN with a larger effective cutoff can be beneficial for very simple systems but requires extensive memory for chemically more diverse systems.

**Figure 4:**
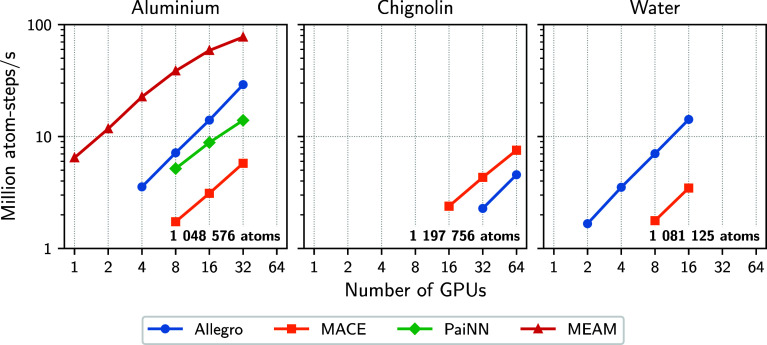
Strong scaling on JEDI for Allegro (blue markers and lines), MACE (orange markers and lines), and PaiNN (green markers and lines) applied to solid state aluminum (fcc) at 1000 K (left), replicated box of solvated Chignolin at ambient conditions (middle), and water slab at ambient conditions (right) for systems with approximately 1 million atoms. The exact numbers of atoms are shown in bold in the lower right corners of the plots. Missing results correspond to simulations that failed due to insufficient memory. Scaling for the modified embedded atom method (MEAM) potential^67^ applied to the aluminum system is shown as a reference (red markers and line).

We additionally compare the scaling with other implementations and models as a reference. For the aluminum system, all models scaled better than a classical MEAM potential^67^ (Figure 4) but were slower for the same number of GPUs. The computational speed of the MEAM potential is naturally higher due to a simpler computation, which affects the model’s accuracy (Table 1). The Allegro models for the aluminum and water system showed strong scaling comparable to Allegro models deployed to similar systems with Allegro-LAMMPS^18,70^ (Supporting Information, Figure S3). However, the exact difference in computational speed depends on the model architecture, such as the depth of the model and the cutoff of the graph.

## 5 Discussion

We present chemtrain-deploy, a model-agnostic framework for deploying JAX-based semilocal MLPs to LAMMPS. By coupling JAX-based models with the scalability and functionality of LAMMPS, chemtrain-deploy provides a seamless interface for running complex and large-scale simulations with minimal integration overhead on multiple GPUs. Therefore, chemtrain-deploy overcomes key limitations of existing software, which are often restricted to specific model architectures, provide limited training support, or face scalability challenges.

Our results demonstrate excellent scaling of modern GNN potentials through chemtrain-deploy for different systems, particularly in simulations involving millions of atoms across multiple GPUs. Through optimizations such as XLA-based compilation and graph pruning, we reduce the execution overhead and minimize recompilation costs. This capability enables the application of semilocal MLPs to new fields in computational biology and material sciences.

We compared different state-of-the-art GNN architectures. We found that strictly local models generally exhibit superior scalability due to their limited communication overhead, while semilocal message-passing GNNs tend to provide improved accuracy but can exhibit reduced scalability and increased memory demands. Nonetheless, actual computational performance depends on the specific MLP hyperparameters and the systems of interest to practitioners. These insights might provide a starting point for future users to select architectures and guide the development of MLPs.

chemtrain-deploy can also support other semilocal MLPs beyond those models demonstrated in this paper, such as Behler-Parinello potentials,^55^ NequIP,^19^ DimeNet++,^71^ and future MLPs likely to be available in a JAX-based implementation. Moreover, these architectures can be combined, e.g., with classical field priors for coarse-grained systems,^33,72^ or Coulomb interactions and dispersion corrections^73^ to form hybrid classical/GNN potentials with high stability and effective treatment of long-range interactions.^36,38–40^ From a future perspective, the flexibility and extensibility of chemtrain-deploy ensure its long-term usability beyond current state-of-the-art models and foster innovation by enabling rapid implementation and testing.

Looking forward, several promising avenues exist to extend the capabilities of chemtrain-deploy. These include support for global models that can capture long-range interactions,^37^ integration with other popular MD software such as GROMACS^28^ and NAMD,^74^ implementation of adaptive cutoff schemes,^70^ and multiscale modeling techniques such as multitime-step algorithms.^75^ Such developments will expand the applicability of chemtrain-deploy to an expanded set of complex systems and simulation scenarios, further bridging the gap between MLPs and practical large-scale molecular simulations.

The broad applicability of chemtrain-deploy opens several exciting opportunities for the MD community. Its ability to efficiently handle million-atom systems enables simulations of complex materials and biological systems. By making use of the various functions of LAMMPS, chemtrain-deploy supports nonstandard simulation protocols such as those under external fields or using enhanced sampling techniques, expanding the range of molecular phenomena that can be studied. Importantly, its model-agnostic design allows for easy benchmarking and comparison of different MLPs within consistent simulation settings, accelerating the development and validation of next-generation models. Together, we expect that chemtrain-deploy will accelerate the adoption and development of advanced MLPs, enabling transformative advances in large-scale molecular simulations and ultimately driving progress across computational chemistry and materials science.

## Supplementary Material



## Data Availability

The ANI-AL data set is available at https://github.com/atomistic-ml/ani-al. The SPICE data set can be accessed at https://github.com/openmm/spice-dataset. The H_2_O-PBE0TS data set is available at https://aissquare.com/datasets/detail?pageType=datasets&name=H2O-PBE0TS. The parameters for the MEAM potential for aluminum can be accessed at https://www.ctcms.nist.gov/potentials/entry/2003--Lee-B-J-Shim-J-H-Baskes-M-I--Al/. The chemtrain framework, including chemtrain-deploy, is open-source and available at https://github.com/tummfm/chemtrain with documentation avnailable at https://chemtrain.readthedocs.io/en/latest/. The LAMMPS molecular dyamics package is publicly available at https://github.com/lammps/lammps. Scripts for model definition, training, and benchmarking are publicly available at https://github.com/tummfm/chemtrain-deploy.

## Notes

Additional information, results, and evaluations, including model hyperparameters, parallel efficiency measurements, and runtime comparisons (PDF)
