# Supplementary material for: chemtrain-deploy: A Parallel and Scalable Framework for Machine Learning Potentials in Million-Atom MD Simulations
Source: J Chem Theory Comput. 2025 Jul 23;21(15):7550–60. doi: 10.1021/acs.jctc.5c00996 (PMC12355694; doi:10.1021/acs.jctc.5c00996)
Supplement: Supplementary file 1 [file ct5c00996_si_001.pdf]

Supplementary Information

`chemtrain-deploy`: A parallel and scalable framework for machine learning potentials in million-atom MD simulations.

Paul Fuchs[1], Weilong Chen[1], Stephan Thaler[3], Julija Zavadlav[1,2*]

[1]Professorship of Multiscale Modeling of Fluid Materials, Department of Engineering Physics and Computation, TUM School of Engineering and Design, Technical University of Munich, Germany.
[2]Atomistic Modeling Center (AMC), Munich Data Science Institute (MDSI), Technical University of Munich, Germany.
[3]Valence Labs, Montreal, QC, Canada.

*Corresponding author(s). E-mail(s): julija.zavadlav@tum.de;

1 Reference Training Datasets

ANI-AL

The dataset includes over 6000 DFT calculations on supercells containing up to 250 atoms, covering a wide range of nonequilibrium configurations. It was generated using a minimally guided active learning approach. The data can be obtained from https://github.com/atomistic-ml/ani-al.

SPICE

SPICE is a quantum chemistry dataset for simulating drug-like small molecules and proteins. It contains over 1.1 million configurations, representing a diverse set of small molecules, dimers, dipeptides, and solvated amino acids. The dataset provides energies and forces computed using the ωB97M-D3(BJ)/def2-TZVPPD level of theory. The data can be obtained from https://github.com/openmm/spice-dataset.

H_2O-PBE0TS

The H_2O-PBE0TS dataset contains snapshots of liquid water and ice configurations generated via ab initio molecular dynamics (AIMD) using the PBE0+TS functional. The data can be obtained at https://aissquare.com/datasets/detail?pageType=datasets&name=H2O-PBE0TS.

2 Model Training

2.1 Training Note

During our experiments, we could achieve reasonable accuracy in terms of energy and force predictions with a moderate number of parameters for the Allegro architecture, but the trained models tended to exhibit instabilities and distortions of the molecular geometries during MD simulations for the SPICE dataset. This dataset contains chemically more diverse and complex samples than the other datasets. Therefore, we mitigated this problem by increasing the embedding dimension, channels of the hidden irreps, and the number of layers. Nevertheless, these adjustments significantly increased the computational cost but only slightly increased the predictive accuracy. In contrast, PaiNN and MACE generally exhibit more robust behavior, maintaining both accuracy and simulation stability across systems with less extensive tuning. These observations emphasize that errors on a test dataset are insufficient to compare models and highlight the importance of testing models under realistic simulation scenarios.

2.2 Hyperparameters

For MACE, the energy and force weights in the loss function are set to $\lambda_E = 10^{-6}$ and $\lambda_F = 10^{-1}$, respectively. For PaiNN, we use $\lambda_E = 10^{-4}$ and $\lambda_F = 10^{-1}$. For Allegro on water and aluminum, the weights are $\lambda_E = 10^{-6}$ and $\lambda_F = 10^{-1}$, while for Chignolin, both are set to $\lambda_E = 10^{-4}$ and $\lambda_F = 10^{-4}$.

For all Allegro models on water and alumnium, we use one tensor product layer with $l_{\max} = 3$, 8 radial basis functions, and a polynomial envelope of order 2, while

we use three layers with $l_{\max} = 2$ for Chignolin. For MACE models, we set the hidden irreducible representations to "$32 \times 0e + 32 \times 1o$" across all cases, with $l_{\max} = 3$, a correlation order of 3 per layer, 2 interaction layers, a node embedding dimension of 64, 8 radial basis functions, and a polynomial envelope of order 6. For PaiNN, we use 4 layers in all cases and vary only the size of the embedding features, keeping all other hyperparameters fixed. The learning rate generally follows a polynomial decay schedule with a power of 2.0 and a decay rate 10^{-5}. The only exception is for the Allegro model on the SPICE dataset, where we use an exponential decay schedule with a decay rate of 0.001.

H_2O-PBE0TS and ANI-AL Models

The H_2O-PBE0TS models were trained on a total of 100,000 samples. For Allegro, we use a hidden MLP layer dimension of 64 and embedding dimensions of [8, 16, 32]. The hidden irreducible representations are set to "$32\times0e+16\times1e+16\times1o+8\times2e+8\times2o$". For both MACE and Allegro, the learning rate is set to 0.01. For PaiNN, we use a hidden feature size of 128 with a initial learning rate of 0.001.

The ANI-AL models were trained on 6,000 samples. All hyperparameters are kept the same as in the H_2O-PBE0TS case, except that the PaiNN hidden feature size is set to 64. The learning rates remain the same, while the batch sizes are 64 for Allegro, 16 for MACE, and 8 for PaiNN.

SPICE Models

We use the entire dataset for training, excluding the Ion Pairs subset, with a total of 1,817,199 samples. For Allegro, the hidden MLP layer dimension is set to 256, with embedding dimensions of [128, 128, 256]. The hidden irreducible representations are set to "$64 \times 1o + 16 \times 2e$". For both Allegro and MACE, the initial learning rate is 0.001, with batch sizes of 16. For PaiNN, we use a hidden feature size of 128, a learning rate of 10^{-4}, and a batch size of 32.

3 Supplementary Results

Table S1: Runtime (in million atom steps/s) for JAX, M.D. and `chemtrain-deploy` on a single A100 GPU (80GB) using Allegro, MACE, and PaiNN architectures. Benchmarks were performed on: solid-state aluminium (FCC) at 1000 K, a replicated solvated Chignolin box at ambient conditions, and a water slab at ambient conditions. System sizes were selected to fit within the memory limits of both JAX, M.D. and `chemtrain-deploy`.

**Architecture**	**System**	**System Size**	**JAX, M.D.**	`chemtrain-deploy`
Allegro	Aluminium	296,352	0.670	0.531
	Chignolin	27,936	0.040	0.037
	Water	253,125	0.767	0.561
MACE	Aluminium	108,000	0.185	0.119
	Chignolin	94,284	0.154	0.088
	Water	112,500	0.150	0.126
PaiNN	Aluminium	171,500	0.857	0.395
	Chignolin	3,492	0.115	0.027
	Water	40,500	0.199	0.119

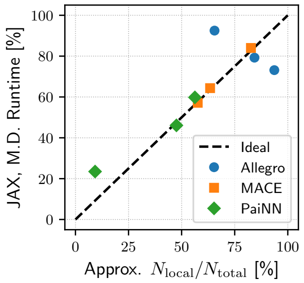

Figure S1: JAX, M.D. runtime relative to `chemtrain-deploy` runtime on a single GPU (A100, 80GB) for Allegro (blue), MACE (orange), and PaiNN (green) applied to solid state aluminium (fcc) at 1000 K (left), replicated box of solvated Chignolin at ambient conditions (middle), and water slab at ambient conditions plotted against the approximate ratio of local atoms to total atoms, i.e., local and copied atoms. The two points above the line correspond to the smallest Chignolin systems with $27,936$ atoms. The point below the line corresponds to the water system with $253,125$ atoms. The black dashed line denotes the idealized runtime of JAX, M.D.

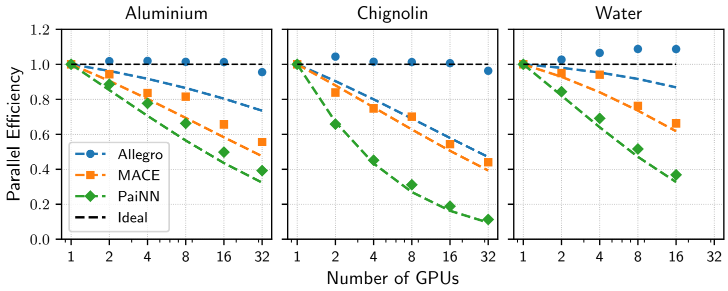

Figure S2: Parallel efficiency on JEDI for Allegro (blue), MACE (orange), and PaiNN (green) applied to **a** solid state aluminium (fcc) at 1000 K, **b** replicated box of solvated Chignolin at ambient conditions, and **c** water slab at ambient conditions. Ideal and approximate (Eq. 4) parallel efficiency are shown as dashed lines in black and the model colors, respectively. The sizes of each system are given in Figure 2 (main paper) as numbers of atoms in the lower right corners of the plots.

Table S2: Simulation speed [ns/day] on JEDI for Allegro, MACE, and PaiNN applied to **a** solid state aluminium (fcc) at 1000 K, **b** replicated box of solvated Chignolin at ambient conditions, and **c** water slab at ambient conditions. Time steps of 3fs, 0.5fs, and 1fs were used to simulate the aluminium, Chignolin, and water systems. Results for the modified embedded atom method (MEAM) potential [1] are shown for the aluminium system and crossed (×) otherwise. Dashes denote simulations that failed due to memory limitations.

System	Atom count	GPU count	MEAM	Allegro	MACE	PaiNN
Aluminium	1,048,576	1	1.60	–	–	–
		2	2.90	–	–	–
		4	5.59	0.88	–	–
		8	9.54	1.77	0.43	1.28
		16	14.53	3.47	0.77	2.19
		32	19.13	7.19	1.43	3.45
Chignolin	1,197,756	16	×	–	0.09	–
		32	×	0.08	0.16	–
		64	×	0.16	0.27	–
Water	1,081,125	2	×	0.13	–	–
		4	×	0.28	–	–
		8	×	0.56	0.14	–
		16	×	1.15	0.28	–

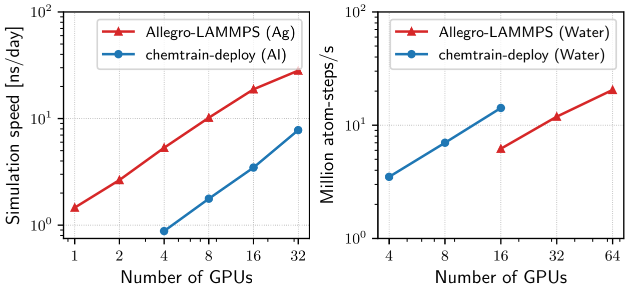

Figure S3: Left: Simulation speed comparison between `chemtrain-deploy` (Aluminum, 3 fs timestep) and `Allegro-LAMMPS` (Silver, 5 fs timestep) across varying numbers of GPUs. This comparison is provided mainly for reference, as our model is more complex. For example, `Allegro-LAMMPS` uses a lightweight setup with a single layer, one even-parity feature, $l_{\max} = 1$, shallow MLPs ([16, 32] and [32]), and a radial cutoff of 4.0 Å. In contrast, our `chemtrain-deploy` model for aluminum employs a more demanding configuration, as detailed in the main paper. Right: Strong scaling results for a fixed one-million-atom water system using `chemtrain-deploy`. Note that the exact hyperparameters used in the `Allegro-LAMMPS` benchmarks were not reported [2, 3].

References

[1] Lee, B.-J., Shim, J.-H., Baskes, M.I.: Semiempirical atomic potentials for the fcc metals cu, ag, au, ni, pd, pt, al, and pb based on first and second nearest-neighbor modified embedded atom method. Phys. Rev. B **68**, 144112 (2003) https://doi.org/10.1103/PhysRevB.68.144112

[2] Musaelian, A., Batzner, S., Johansson, A., Sun, L., Owen, C.J., Kornbluth, M., Kozinsky, B.: Learning local equivariant representations for large-scale atomistic dynamics. Nature Communications **14**(1), 579 (2023) https://doi.org/10.1038/s41467-023-36329-y

[3] Kozinsky, B., Musaelian, A., Johansson, A., Batzner, S.: Scaling the leading accuracy of deep equivariant models to biomolecular simulations of realistic size. In: Proceedings of the International Conference for High Performance Computing, Networking, Storage and Analysis. Sc '23. Association for Computing Machinery, New York, NY, USA (2023). https://doi.org/10.1145/3581784.3627041
